# Seed dormancy back on track; its definition and regulation by DOG1

**DOI:** 10.1111/nph.16592

**Published:** 2020-04-29

**Authors:** Wim J. J. Soppe, Leónie Bentsink

**Affiliations:** ^1^ Rijk Zwaan De Lier 2678 ZG the Netherlands; ^2^ Wageningen Seed Science Centre Laboratory of Plant Physiology Wageningen University 6708 PB Wageningen the Netherlands

**Keywords:** DELAY OF GERMINATION 1, DSDS50, germination rate, seed dormancy, T50

Seed traits like dormancy, germination rate, and longevity determine the start of the life cycle of plants. The desired values for these traits can be very different for plants growing under natural or agricultural conditions. For the first, germination should be postponed until the start of a favorable growth season and germination should be well balanced with the prevailing environmental conditions. For crop plants, germination should be immediate, uniform and fast.

Dormancy is defined as the absence of germination of a viable seed under conditions that are suitable for germination. Dormancy is usually measured on a population of seeds. Seed populations that do not fully germinate in conditions that favor germination can therefore be described as having a certain level of dormancy. Reduction in dormancy leads to a widening of the environmental conditions at which a seed can germinate and often to an increase in the rate of germination. However, we would advocate not to mix up germination rate with seed dormancy as we sometimes see in comparisons of mutant and wild‐type seed batches.

Dormancy should be determined by analyzing germination under suitable conditions. Absence of germination or low germination percentages in populations of seeds indicate dormancy, which can be overcome either by extended storage in dry conditions (after‐ripening) or imbibition at specific low or high temperatures (depending on the plant species; stratification). These dormancy releasing treatments should lead to an increased germination percentage. A typical experiment that allows identification of differences in dormancy level is a so‐called after‐ripening experiment. For this, seeds should be harvested from plants that are grown at the same time in the same conditions. Germination experiments are subsequently performed at increasing time intervals during seed dry storage. Generally it is seen that both germination percentage and germination rate increase with longer storage times (Ni & Bradford, 1993; Alvarado & Bradford, 2005; Meyer & Allen, 2009; Batlla & Benech‐Arnold, 2015). To determine dormancy differences it is not required to evaluate germination percentage at multiple time‐points after sowing, end‐point measurements are sufficient. The level of dormancy can be expressed in one value by calculating the Days of Seed Dry Storage required to reach 50% of germination (DSDS50 or AR50) (Alonso‐Blanco *et al.*, 2003) (Fig. 1a–d). Alternatively, a germination experiment at a fixed time‐point after seed harvest also allows the identification of differences in dormancy levels (Fig. 1e,f). In such an experiment the seeds should be allowed enough time to germinate to ensure that a plateau phase is reached in order to avoid misinterpretation of differences in germination rate as dormancy. Apart from increasing after‐ripening time, dormancy levels could also be determined by increasing stratification time (Kendall *et al.*, 2011).

**Fig. 1:**
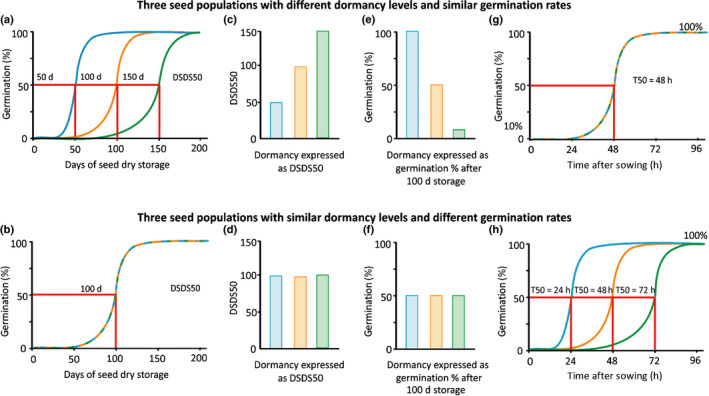
Schematic presentation of graphs resulting from two different hypothetical germination experiments that allow the identification of differences in dormancy and germination rate. (a, b) These graphs represent typical after‐ripening curves, in which the maximum germination capacity of seed batches is determined at different time points during seed dry storage. These graphs allow determining differences in dormancy level. The longer the seeds are stored the more dormancy is released and the higher the germination percentage. Low dormant seed batches reach their full germination capacity after shorter storage times compared to high dormant seed batches. These after‐ripening curves allow the calculation of the DSDS50 (Days of Seed Dry Storage required to reach 50% of germination); indicated by the red lines. Seed batches with higher dormancy levels have higher DSDS50 values. (c, d) Graphs representing the DSDS50 for the seed batches shown in (a) and (b). (e, f) These figures show the germination level as determined at a single time point, for the seed batches presented in (a) and (b). Seed batches with higher dormancy have lower germination percentages. (g, h) Graphs representing the progression of germination over time of the same seed batches as in (a) and (b) after 200 d of seed dry storage, allowing the identification of differences in germination rate. The germination percentage is determined at different time points after sowing. Seed batches with a high germination rate reach their maximum germination earlier then seed batches that have a lower germination rate. Graphs (a, c, e, g) show seed batches with different dormancy levels, the rate of germination in these samples is equal, all have a T50 (time in which 50% of the maximum germination percentage is reached) of 48 h. Graphs (b, d, f, h) represent seed batches with equal dormancy levels, however the relative rate of germination (T50) in these batches differs; indicated by the red lines. All graphs show three hypothetical populations, which are overlapping in (b) and (g).

Differences in germination rate can be caused by genetic differences, as a response to the environment during seed production, or by the interaction between these two. This can often be observed in seed batches of crop plants (Li *et al.*, 2011; Yang *et al.*, 2015; Shim *et al.*, 2020). Mutants that affect the sensitivity to environmental factors for germination can also show altered germination rates (Gualberti *et al.*, 2002; Yu *et al.*, 2019). Finally, germination rate can be associated with seed viability and aged seeds generally need more time to germinate (Bewley *et al.*, 2013). However, differences in germination rate should not be confused with dormancy. To identify differences in germination rate, germination needs to be investigated at multiple time‐points after sowing. Preferably such analyses should be performed in fully after‐ripened seeds, since only then an effect of residual dormancy can be excluded. Germination rates can be visualized in graphs showing germination percentage on the *y*‐axis and imbibition time (or time after sowing) on the *x*‐axis. From such graphs the T50, which is the time in which half of the maximum germination percentage is reached, can be calculated (Fig. 1g,h). As germination rate can be derived from these graphs it is inappropriate to label the *y*‐axis with ‘germination rate’ instead of ‘germination percentage’. We see this frequently in the literature and would like to appeal to editors of scientific journals to prevent the interchange of germination ‘percentage’ and ‘rate’ as if they were identical.

Seed dormancy is an adaptive trait, which is regulated by endogenous and environmental factors. These factors influence the levels of central dormancy regulators that, in their turn, control downstream mechanisms enabling the binary decision of a seed to germinate or not. Our current view is that the hormone abscisic acid (ABA) and the dormancy protein DELAY OF GERMINATION 1 (DOG1) are two of the most important central regulators of seed dormancy in Arabidopsis, as well as in other studied plant species. Both factors are necessary for seed dormancy and the absence of a single one of them leads to loss of dormancy. Several excellent recent reviews have extensively described the roles of these and other factors in dormancy (Chahtane *et al.*, 2017; Finch‐Savage & Footitt, 2017; Nonogaki, 2019). The intention of this Letter is not to repeat these reviews but to focus on the function of DOG1 because, in our opinion, recent publications did not always reflect the latest insights. *DOG1* was originally identified as a seed dormancy quantitative trait locus (QTL) in Arabidopsis (Alonso‐Blanco *et al.*, 2003). The *DOG1* gene was cloned 14 years ago and encodes a protein that lacks domains with a known function, which made it very difficult to obtain clues about its mode of action. In the first paper that described the cloning of the *DOG1* gene, it was suggested that DOG1 might have a direct role in transcriptional regulation based on a low level of homology to the wheat transcription factor HBP‐1b. In addition, one of the three protein domains of DOG1 is also present in group D bZIP transcription factors, although other conserved domains of the bZIP proteins are missing (Bentsink *et al.*, 2006). In the first 10 years after its cloning, no clear insights into its function were obtained and no confirmation for a role of DOG1 as a transcriptional factor was found. Nevertheless, it has been shown that the transcriptome of nondormant seeds discriminates already shortly after rehydration from that of dormant seeds (Dekkers *et al.*, 2016, 2016a,b). Accordingly, seeds of the *dog1* mutant showed altered transcription of hundreds of genes (Dekkers *et al.*, 2016, 2016a,b). We think it is obvious that a mutant that has a strong impact on the dormancy level of seeds also causes major transcriptional changes, but this does not directly imply that it actually functions as a transcription factor.

The conserved and important role of DOG1 in seed dormancy was confirmed in various plant species like wheat (Ashikawa *et al.*, 2014) and lettuce (Huo *et al.*, 2016), and its function in several dormancy‐related processes was revealed. This showed that DOG1 influences processes that are related to the regulation of germination and dormancy like endosperm weakening (Graeber *et al.*, 2014) and controlling the level of microRNAs involved in phase transition (Huo *et al.*, 2016). More direct insights into the mechanism by which DOG1 influences dormancy and germination were obtained recently when two laboratories independently showed that the DOG1 protein binds to and inhibits the action of specific members of clade A PP2C phosphatases in seeds. A genetic analysis showed that two of these phosphatases, ABA‐HYPERSENSITIVE GERMINATION 1 (AHG1) and AHG3 are necessary for the function of DOG1 (Née *et al.,* 2017; Nishimura *et al.*, 2018). Interestingly, this group of phosphatases also has a central role in ABA signaling and therefore represents a converging point of these two important regulators of dormancy (Fig. 2). A protein that functions by inhibiting phosphatases is unlikely to have a direct role in transcriptional regulation. Therefore, the first ideas about the function of DOG1 as a transcriptional factor are unlikely to be true. Further studies are required to identify the functional domains of DOG1, their exact molecular roles and downstream (transcription) factor(s).

**Fig. 2:**
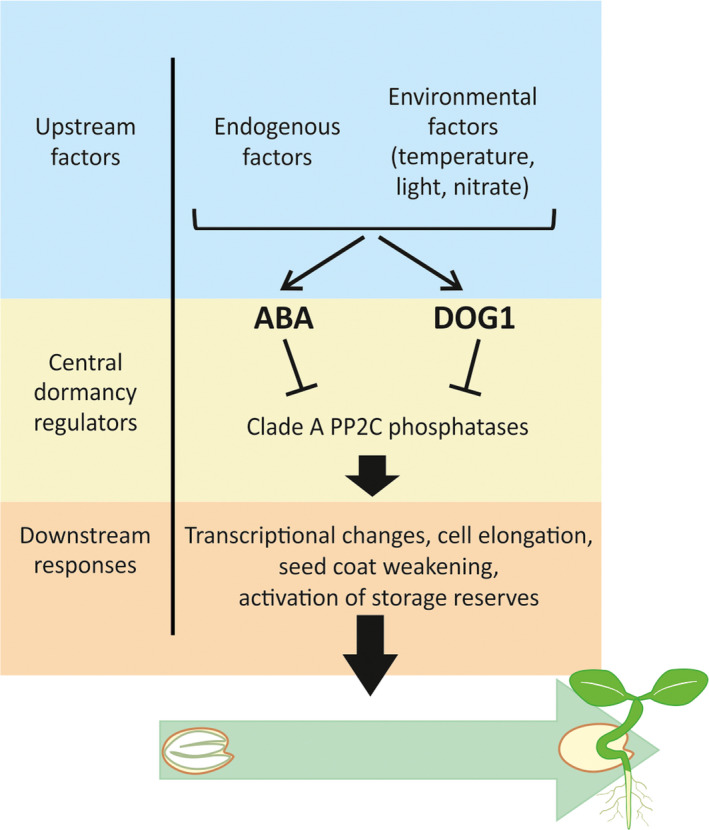
An overview of the regulation of germination by the central dormancy regulators abscisic acid (ABA) and DELAY OF GERMINATION 1 (DOG1). The levels of ABA and DOG1 are influenced by upstream endogenous and environmental factors. ABA and DOG1 function by inhibiting the action of PP2C clade A phosphatases, which regulate downstream responses that determine whether a seed will germinate or not. Arrows illustrate activating and blunt ended lines inhibiting effects.

With this Letter, we would like to advocate for a correct use of the terms seed dormancy and germination rate and for a proper reference to the latest insights of the actions of its most important regulators. We think that this is necessary to focus dormancy research in the right direction and to avoid following outdated leads.

## Author contributions

WJJS and LB wrote the manuscript. WJJS and LB contributed equally to this work.
